# In Vitro Evaluation of the Squaramide-Conjugated Fibroblast Activation Protein Inhibitor-Based Agents AAZTA^5^.SA.FAPi and DOTA.SA.FAPi

**DOI:** 10.3390/molecules26123482

**Published:** 2021-06-08

**Authors:** Euy Sung Moon, Yentl Van Rymenant, Sandeep Battan, Joni De Loose, An Bracke, Pieter Van der Veken, Ingrid De Meester, Frank Rösch

**Affiliations:** 1 Department of Chemistry–TRIGA, Johannes Gutenberg University Mainz, 55128 Mainz, Germany; emoon01@uni-mainz.de (E.S.M.); sbattan@students.uni-mainz.de (S.B.); 2 Laboratory of Medical Biochemistry, Department of Pharmaceutical Sciences, University of Antwerp, 2610 Wilrijk, Belgium; Yentl.VanRymenant@uantwerpen.be (Y.V.R.); joni.deloose@uantwerpen.be (J.D.L.); an.bracke@uantwerpen.be (A.B.); ingrid.demeester@uantwerpen.be (I.D.M.); 3 Laboratory of Medicinal Chemistry, Department of Pharmaceutical Sciences, University of Antwerp, 2610 Wilrijk, Belgium; pieter.vanderveken@uantwerpen.be

**Keywords:** AAZTA, scandium-44, lutetium-177, FAP, SA, DPP, PREP

## Abstract

Recently, the first squaramide-(SA) containing FAP inhibitor-derived radiotracers were introduced. DATA^5m^.SA.FAPi and DOTA.SA.FAPi with their non-radioactive complexes showed high affinity and selectivity for FAP. After a successful preclinical study with [^68^Ga]Ga-DOTA.SA.FAPi, the first patient studies were realized for both compounds. Here, we present a new squaramide-containing compound targeting FAP, based on the AAZTA^5^ chelator 1,4-bis-(carboxylmethyl)-6-[bis-(carboxymethyl)-amino-6-pentanoic-acid]-perhydro-1,4-diazepine. For this molecule (AAZTA^5^.SA.FAPi), complexation with radionuclides such as gallium-68, scandium-44, and lutetium-177 was investigated, and the in vitro properties of the complexes were characterized and compared with those of DOTA.SA.FAPi. AAZTA^5^.SA.FAPi and its derivatives labelled with non-radioactive isotopes demonstrated similar excellent inhibitory potencies compared to the previously published SA.FAPi ligands, i.e., sub-nanomolar IC_50_ values for FAP and high selectivity indices over the serine proteases PREP and DPPs. Labeling with all three radiometals was easier and faster with AAZTA^5^.SA.FAPi compared to the corresponding DOTA analogue at ambient temperature. Especially, scandium-44 labeling with the AAZTA derivative resulted in higher specific activities. Both DOTA.SA.FAPi and AAZTA^5^.SA.FAPi showed sufficiently high stability in different media. Therefore, these FAP inhibitor agents could be promising for theranostic approaches targeting FAP.

## 1. Introduction

Fibroblast activation protein (FAP) is a post-prolyl proteolytic enzyme that belongs to the S9 family of serine proteases [1]. In addition to FAP, this S9 family includes other proline-specific serine proteases, such as prolyl oligopeptidase (PREP) and the dipeptidyl peptidases 4, 8, and 9 (DPP4, DPP8, and DPP9). Targeting fibroblast activation protein (FAP), overexpressed selectively in cancer-associated fibroblasts (CAFs), has recently become an attractive goal for diagnostic imaging and first therapeutic trials. FAP is involved in the promotion and development of tumor growth and is typically overexpressed in activated fibroblasts in the tumor stroma, whereas it is absent in most normal healthy tissues. Furthermore, FAP is overexpressed in several pathological tissue sites that are characterized by active remodeling [2,3,4,5]. Expression of FAP is found in CAFs in approximately 90% of epithelial carcinomas such as breast, pancreatic, colon, and prostate tumors [6,7,8]. These properties make FAP a very interesting and universally applicable tumor target for a variety of tumor types.

PET tracers that operate as FAP-specific enzyme inhibitors (FAPi), have first been published by Lindner and Loktev et al. [9,10,11]. The FAP inhibitor used is a small molecule with an N-acylated glycyl(2-cyano-4,4-difluoropyrrolidine) that binds to FAP active site and blocks its enzymatic activity. This highly potent inhibitor, referred to as UAMC1110, shows high affinity for FAP but not for the DPPs and PREP [12]. Meanwhile, many clinical trials have been initiated with related PET tracers based on the same FAP inhibitor [13,14,15,16,17,18,19,20,21]. Lindner and Loktev et al. developed DOTA-based FAP inhibitor conjugates with heterocyclic units as spacer. The most prominent are FAPI-04 and FAPI-46, with piperazine between chelator and inhibitor. Other examples include a glycosylated fluorine-18 derivative and tracers for SPECT applications with technetium-99 and rhenium-188 introduced by tricarbonyl ligands with piperazine linker systems [22,23]. Recently, we developed FAP inhibitor agents using squaramide-combined bifunctional chelators [24]. The use of a squaramide linker facilitated the synthetic work and delivered compounds with good pharmacological properties. The latter results were illustrated by the excellent in vitro affinity of these products for FAP and by their in vivo behavior in preclinical and clinical applications. With respect to clinical studies, the DATA^5m^.SA.FAPi derivative showed specific tracer uptake in focal nodular hyperplasia via ^68^Ga-PET/CT [25]. The DOTA.SA.FAPi tracer displayed a high target-to-background ratio during ^68^Ga-PET/CT studies in patients with various cancers [26].

The advantageous properties of gallium-68, such as its high positron energy with β^+^ = 89% and E_β,avg_ = 830 keV and its good accessibility due to the availability of ^68^Ge/^68^Ga generators, make it a commonly used PET radionuclide [27]. However, the short physical half-life of the nuclide (1.1 h) may impede focusing on longer-lasting physiological processes in PET/CT measurements. Scandium-44, which is also characterized by a high branching ratio of β^+^ = 94% and E_β,avg_ = 632 keV, could be a valuable alternative with a t_1/2_ of 4.0 h. There are two ways to produce scandium-44: one uses a cyclotron via the ^44^Ca(p,n)^44^Sc reaction, and the other, that we chose, uses a ^44^Ti/^44^Sc generator [27,28,29]. An established post-processing elution protocol provides carrier-free scandium-44 from a 185 MBq generator, with ~90% elution efficiency and a titanium-44 breakthrough of only <7 MBq [30]. Due to its four-time longer half-life than gallium-68, the β^+^-emitter scandium-44 could be better suited for pretherapeutic PET/CT measurements resulting in individual dosimetric calculations in endoradiotherapy with longer-lived therapy nuclides such as yttrium-90, lutetium-177, or scandium-47. Scandium-44 has already been used in both preclinical and clinical applications [31,32,33,34,35]. In particular, the first in human PET measurements in metastasized castrate-resistant prostate cancer with [^44^Sc]Sc-PSMA-617 indicated its potential as PET nuclide and pre-therapeutic agent [36]. Furthermore, the β^-^-emitter lutetium-177, with a half-life of 6.7 days, is nowadays a very commonly used radionuclide in radioendotherapy. It is clinically used for neuroendocrine tumors in the somatostatin analogues [^177^Lu]Lu-DOTATOC and [^177^Lu]Lu-DOTATATE for peptide-mediated radioreceptor therapy and for treatment of prostate carcinomas by means of lutetium-177-PSMA therapy with PSMA derivatives such as PSMA-617 and PSMA-I&T [37,38,39,40,41,42,43].

In this work, we introduce a novel FAP inhibitor agent called AAZTA^5^.SA.FAPi. AAZTA chelators allow fast and quantitative complexation under mild conditions and display high stability. This is in particular relevant for radionuclides with high needs of coordination capacity, such as scandium-44 and lutetium-177.

Together with the recently published DOTA.SA.FAPi, both precursors were radiochemically investigated in terms of labeling and stability with gallium-68, scandium-44, and lutetium-177 and tested for their in vitro properties.

## 2. Results and Discussion

### 2.1. Synthesis of Chelator Conjugates

**Figure 1:**
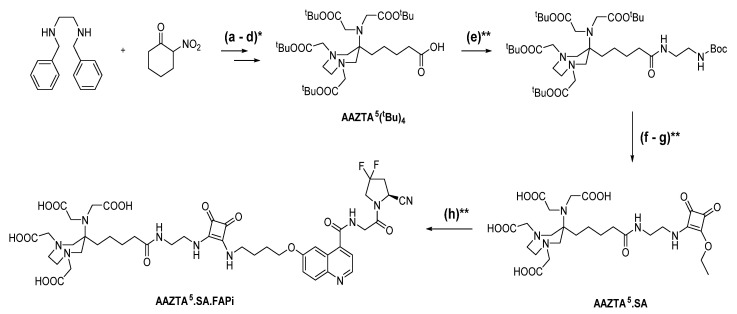
Synthesis of AAZTA^5^.SA.FAPi via AAZTA^5^(^t^Bu)_4_ and AAZTA^5^.SA: (**a**) 2-nitrocyclohexanone, Amberlyst A21, paraformaldehyde, methanol, 80 °C, 16 h; (**b**) palladium hydroxide/C, acetic acid, hydrogen, ethanol, 25 °C, 16 h; (**c**) tert-butyl bromoacetate, potassium carbonate, potassium iodide, acetonitrile, 40 °C, 48-72 h; (**d**) 1 M lithium hydroxide, 1,4-dioxane/water (2:1), 25 °C, 16 h; (**e**) N-Boc-ethylenediamine, HATU, HOBt, DIPEA, acetonitrile, 25 °C, 16 h; (**f**) dichloromethane/TFA (80:20)%, 25 °C, 7 h; (**g**) 3,4-diethoxycyclobut-3-ene-1,2-dione, 0.5 M phosphate buffer pH = 7, 25 °C, 16 h; (**h**) NH_2_-UAMC1110, 0.5 M phosphate buffer pH = 9, 25 °C, 16 h; (*) as reported [44,45]; (**) as reported [24]. DOTA.SA.FAPi was synthesized as previously described [24].

For AAZTA^5^.SA.FAPi, we first synthesized AAZTA^5^(^t^Bu)_4_. The coupling of squaric acid to the terminal carboxyl group and the subsequent binding to the FAP inhibitor was performed in the same way as for the previously described DATA^5m^.SA.FAPi [24]. Figure 1 shows the synthesis route of AAZTA^5^.SA.FAPi, following the protocol of Sinnes et al. and Greifenstein et al. [44,45].

**Figure 2:**
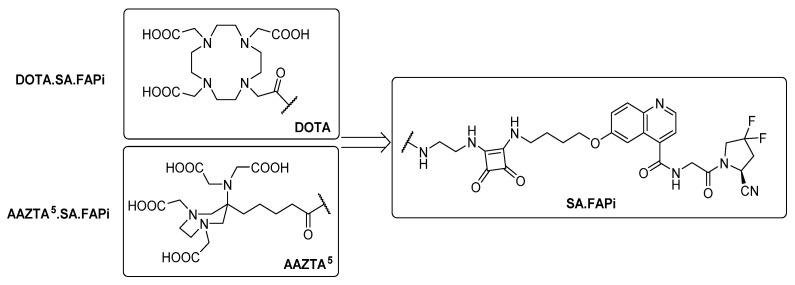
Structures of DOTA.SA.FAPi and AAZTA^5^.SA.FAPi.

Figure 2 shows the structures of the FAP inhibitor probes DOTA.SA.FAPi and AAZTA^5^.SA.FAPi.

### 2.2. In Vitro Inhibition Measurements

The IC_50_ values for FAP, PREP, and the DPPs of the hybrid chelator conjugate AAZTA^5^.SA.FAPi compared to those of DOTA.SA.FAPi are shown in Table 1. The IC_50_ values of AAZTA^5^.SA.FAPi as well as those of its non-radioactive complexes [^nat^Sc]Sc-AAZTA^5^.SA.FAPi and [^nat^Lu]Lu-AAZTA^5^.SA.FAPi for FAP appeared to be in the low nanomolar range (0.55–0.57 nM), whereas the IC_50_ values for PREP resulted in the low micromolar range (2.4–3.6 µM). Screening against DPP4 and DPP9 for both SA.FAPi complexes revealed that the remaining activity was more than 50% at a final concentration of 1 µM. Hence, the IC_50_ values for the DPPs were reported as >1 µM. The absence of a basic amine in the FAP inhibitor is known to result in an enormous increase of selectivity for the target molecule FAP, whereas the affinity for the DPPs can be drastically reduced. [12,46]. The IC_50_ values for FAP and PREP were in the same order of magnitude of those for the previously reported SA.FAPi compounds, i.e., indicating high inhibition potency and excellent FAP-to-PREP selectivity indices. In addition, high selectivity towards DPP4 and DPP9 was achieved.

### 2.3. Radiolabeling and In Vitro Stability in Complexwith Gallium-68, Scandium-44, and Lutetium-177

**Figure 3:**
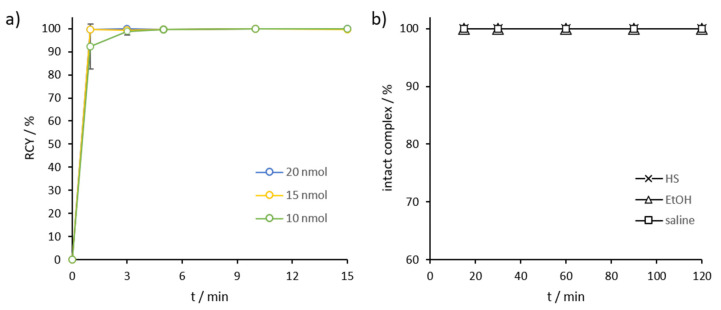
(**a**) Kinetics of [^68^Ga]Ga-AAZTA^5^.SA.FAPi at RT for tracer amounts ≥10 nmol (*n* = 3); (**b**) Stability of [^68^Ga]Ga-AAZTA^5^.SA.FAPi at 37 °C in HS, EtOH, and NaCl over a period of 120 min (*n* = 3).

*Gallium-68*: DOTA.SA.FAPi complexed with gallium-68 showed very high kinetics in quantitative radiochemical yields (RCYs) in our previous work [24]. Gallium labeling of AAZTA^5^.SA.FAPi with diverse precursor amounts (10, 15 and 20 nmol) was performed at room temperature (Figure 3a). [^68^Ga]Ga-AAZTA^5^.SA.FAPi displayed quantitative complexation already after 3–5 min (Figure 3a, Figure S1, Supplementary Material). Compared to the DOTA derivative, complexation led to very high RCYs for tracer amounts ≥10 nmol, even at ambient temperature. In the case of the previously reported DOTA.SA.FAPi, high RCYs could only be achieved with an amount ≥15 nmol and at a high temperature of 95 °C. the stability of [^68^Ga]Ga-AAZTA^5^.SA.FAPi in human serum (HS), ethanol (EtOH), and saline (NaCl) was excellent, with >99.9% intact complexes over a measured time period of 2 h (Figure 3b, Figures S2–S4).

**Figure 4:**
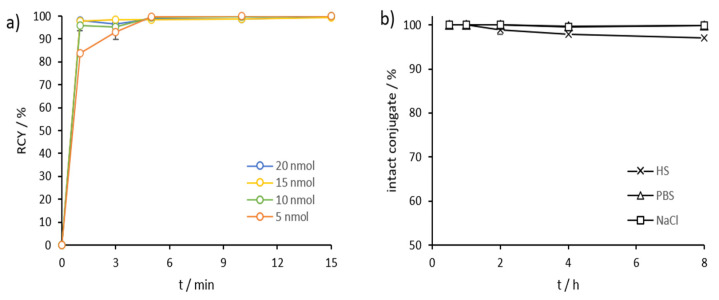
(**a**) Kinetics of [^44^Sc]Sc-AAZTA^5^.SA.FAPi at RT for tracer amounts ≥5 nmol (*n* = 3 for 10; *n* = 1 for 5, 15, and 20 nmol); (**b**) Stability of [^44^Sc]Sc-AAZTA^5^.SA.FAPi at 37 °C in HS, PBS, and NaCl over a period of 8 h (*n* = 3).

*Scandium-44*: AAZTA^5^.SA.FAPi demonstrated excellent complexation with scandium-44 even at RT. We tested 5–20 nmol of precursor, which resulted in quantitative labeling already after 5 min for all amounts (Figure 4a, Figure S5). Stability was tested in HS, phosphate-buffered saline (PBS), and NaCl at 37 °C, demonstrating in highly satisfactory values in all three media (Figure 4b, Figures S6–S11). After 1 h, [^44^Sc]Sc-AAZTA^5^.SA.FAPi conjugates were stable, with >99% intact conjugate in all three media. Even up to the end of the measurement (8 h), the intact conjugates were stable in PBS and saline (>99%) and in HS (>97%) (Figure 4b).

**Figure 5:**
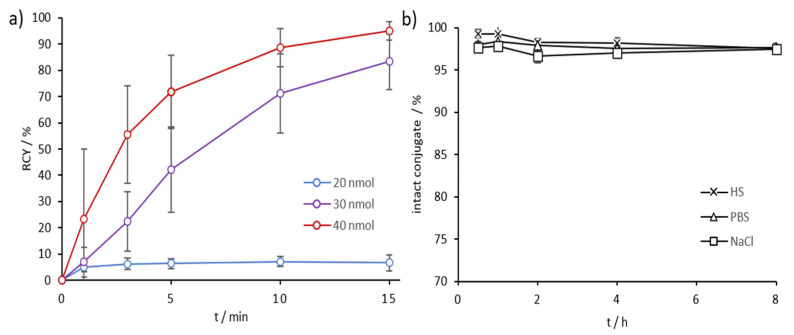
(**a**) Kinetics of [^44^Sc]Sc-DOTA.SA.FAPi at 95 °C for tracer amounts ≥20 nmol (*n* = 5 for 20–40 nmol); (**b**) Stability of [^44^Sc]Sc-DOTA.SA.FAPi at 37 °C in HS, PBS, and NaCl over a period of 8 h (*n* = 3).

DOTA.SA.FAPi also showed good complexation with scandium-44. However, whereas [^44^Sc]Sc-AAZTA^5^.SA.FAPi already displayed quantitative RCYs with 5 nmol (GBq/0.17 µmol) of precursors, [^44^Sc]Sc-DOTA.SA.FAPi showed very low complexation with 20 nmol. Only with a quantity of 30 nmol, DOTA.SA.FAPi high yields with scandium-44 were reached, with RCYs >83% and >95%, when, respectively, 30 and 40 nmol (GBq/1.33 µmol) were used (Figure 5a). Stability in HS, PBS, and NaCl were high over the measured period of 8 h, resulting in >97% intact complexes with ^44^Sc in all three medias (Figure 5b).

**Figure 6:**
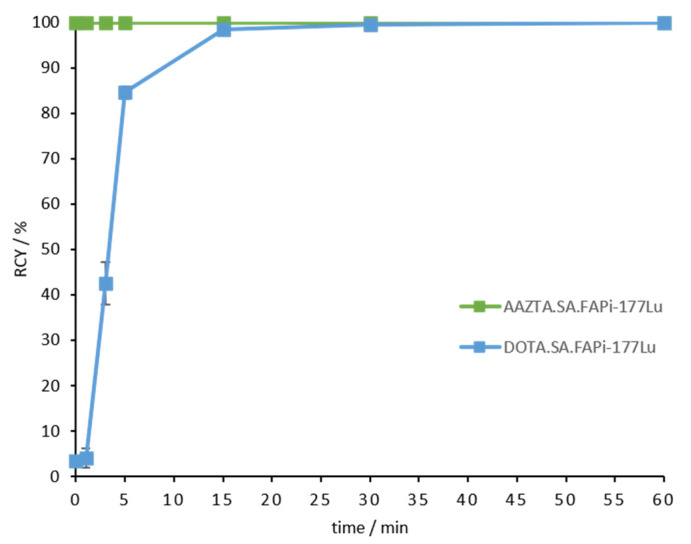
Kinetic measurements for [^177^Lu]Lu-AAZTA^5^.SA.FAPi up to 60 min (green); Kinetic measurements for [^177^Lu]Lu-DOTA.SA.FAPi up to 60 min (blue); (*n* = 3, 20 nmol for both conjugates).

*Lutetium-177*: For both DOTA.SA.FAPi and AAZTA^5^.SA.FAPi, precursors at a concentration of 20 nmol were used for labeling with lutetium-177. Both derivatives presented quantitative complexations with the radiometal after 60 min (Figure 6, Figure S12). The ^177^Lu-AAZTA derivative showed >99% RCY already after 1 min at RT, whereas the ^177^Lu-DOTA derivative reached >99% complexation after 15 min at 95 °C (Figure 6).

**Figure 7:**
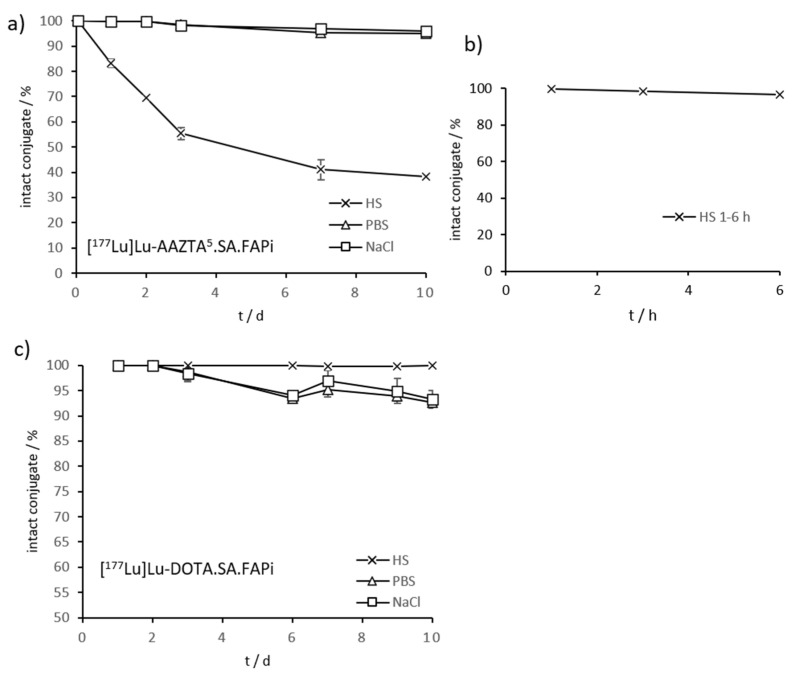
(**a**) Stability of [^177^Lu]Lu-AAZTA^5^.SA.FAPi at 37 °C in HS, PBS, and NaCl over a period of 10 d (*n* = 3); (**b**) Stability of [^177^Lu]Lu-AAZTA^5^.SA.FAPi at 37 °C in HS after 1, 3, and 6 h (*n* = 3); (**c**) Stability measurements of [^177^Lu]Lu-DOTA.SA.FAPi at 37 °C in HS, PBS, and NaCl during 10 d (*n* = 3).

Stability studies of both conjugates were performed in HS, PBS, and saline over a period of 10 days at 37 °C. In PBS and NaCl, very high stability values could be achieved for [^177^Lu]Lu-AAZTA^5^.SA.FAPi, with >99% after 2 d, >98% after 3 d, and >95% intact conjugates after 10 days. In HS, the ^177^Lu-AAZTA complex showed >99% of stability after 1 h, >98% after 3 h, and >96% after 6 h. However, the stability decreased significantly with time. After 1 d, the remaining stability of [^177^Lu]Lu-AAZTA^5^.SA.FAPi in HS was >83%, after 2 d it was >64%, and after 3 d it was >55% (Figure 7a). Nevertheless, the stability of [^177^Lu]Lu-AAZTA^5^.SA.FAPi in HS was satisfactory, with >95% intact conjugate after 6 h. If it is assumed that small molecules accumulate in the target tissue within the first few hours, and therefore their stability in HS over a long period is not relevant. [^177^Lu]Lu-DOTA.SA.FAPi showed very high stability, with >99% of intact conjugate in HS within the measured time period of 10 days. In PBS and NaCl, the stability was high, i.e., >98% after 3 d and still >93% after 10 d (Figure 7b).

### 2.4. Lipophilicity Measurements

Lipophilicity (logD value) was determined via the “shake-flask” method. For both precursors AAZTA^5^.SA.FAPi and DOTA.SA.FAPi logD (pH = 7.4), values were measured for the ^68^Ga- and ^44^Sc complexes. Table 2 shows the logD values for the respective radiotracers.

The lipophilicity of the radiolabeled compounds [^68^Ga]Ga-AAZTA^5^.SA.FAPi, [^68^Ga]Ga-AAZTA^5^.SA.FAPi and [^44^Sc]Sc-AAZTA^5^.SA.FAPi resulted located in hydrophilic ranges. Both gallium-68 derivatives [^68^Ga]Ga-DOTA.SA.FAPi and [^68^Ga]Ga-AAZTA^5^.SA.FAPi showed almost identical logD values of −2.68 and −2.53, respectively. The carboxyl groups and the ionic bonds between chelator and radiometal favor the hydrophilic character of these radiotracers. The logD value of FAPI-04 is reported in the literature as −2.4 ± 0.28, confirming the hydrophilic character of ^68^Ga-DOTA complexes [22]. [^44^Sc]Sc-AAZTA^5^.SA.FAPi display a similar logD value of −2.50 compared to gallium-68 derivatives. There seems to be no great influence of the DOTA and AAZTA chelators in the presence of gallium-68 and scandium-44 radiometals on the lipophilicity of the FAPi radiopharmaceuticals.

## 3. Materials and Methods

### 3.1. General

All basic chemicals were purchased from Merck KGaA (Darmstadt, Germany), TCI Deutschland GmbH (Eschborn, Germany), Fisher Scientific GmbH (Schwerte, Germany), Thermo Fisher GmbH (Kandel, Germany) and VWR International GmbH (Darmstadt, Germany) and used without further purification. (*S*)-6-(4-aminobutoxy)-*N*-(2-(2-cyano-4,4-difluoropyrrolidin-1-yl)-2-oxoethyl)-quinoline-4-carboxamide (called NH_2_-UAMC1110) was purchased from KE Biochem Co. (Shanghai, China). Thin-layer chromatography was performed with silica gel 60 F254-coated aluminum plates that were acquired from Merck KGaA (Darmstadt, Germany). Detection was carried out by fluorescence extinction at λ = 254 nm and by staining with potassium permanganate. The LC/MS spectra were measured on an Agilent Technologies 1220 Infinity LC system coupled to an Agilent Technologies 6130B Single Quadrupole LC/MS system. NMR measurements were performed at 400 MHz (400 MHz FT NMR spectrometer AC 400, Bruker Analytik GmbH). For HPLC (high-performance liquid chromatography) a 7000 series Hitachi LaChrom with a Hitachi L7100 pump, an L7400 UV detector, and a Phenomenex Synergi C18 (250 × 10 mm, 4 µm) column (Aschaffenburg, Germany) were used.

### 3.2. Organic Synthesis

Synthesis of DOTA.SA.FAPi was reported recently [24]. Synthesis of AAZTA^5^.SA.FAPi was realized by first generating AAZTA^5^(^t^Bu)_4_ according to the procedure by Sinnes et al. and Greifenstein et al. [44,45]. Subsequent coupling to the SA.FAPi conjugate was performed using the protocol published earlier for the analogous DATA^5m^.SA.FAPi precursor [24]. After HPLC purification with a gradient of 10–20%, MeCN (+0.1% TFA)/90–80% water (+0.1% TFA) in 20 min, AAZTA^5^.SA.FAPi was obtained as a yellowish solid (16.8 mg; 0.02 mmol; 41%). MS (ESI^+^): 500.3 (M+2H^2+^); 999.3 (M+H^+^); calculated for C_45_H_56_F_2_N_10_O_14_: 998.40.

### 3.3. Non-Radioactive Compounds and In Vitro Inhibition Studies

^nat^Sc/^nat^Lu-AAZTA^5^.SA.FAPi were synthesized by reaction of 5.0 mg (5 µmol) AAZTA^5^.SA.FAPi with 1.5 eq ^nat^ScCl_3_ and ^nat^LuCl_3_, respectively, in 500 µL 0.5 M NaAc buffer pH 4.5 for 2 h at room temperature. Complexation was confirmed by ESI–MS, and HPLC purification was performed with a flow rate 5 mL/min, H_2_O (+0.1% TFA)/MeCN (+0.1% TFA), with a linear gradient condition of 5–95% MeCN in 10 min. The products (4.1 mg; 3.9 µmol; 79% for ^nat^Sc-complex and 4.2 mg; 3.6 µmol; 72%) were obtained as a yellowish powder. MS (ESI^+^) for ^nat^Sc-complex: *m*/*z* (%): 521.3 (M+2H)^2+^; 1041.4 (M+H)^+^ calculated for C_45_H_52_F_2_ScN_10_O_14_: 1039.9 and ^nat^Lu-complex: *m*/*z* (%): 586.2 (M+2H)^2+^; 1171.4 (M+H)^+^ calculated for C_45_H_52_F_2_LuN_10_O_14_: 1169.9.

### 3.4. Inhibition Assays

Enzymes: Recombinant human FAP and PREP were expressed and purified as published [24]. Recombinant human dipeptidyl 9 (DPP9) was purified as described by De Decker et al. [46]. Human dipeptidyl peptidase 4 was purified from seminal plasma as published [47].

IC_50_ measurements and counter-screening: IC_50_-measurements of the probes for FAP and PREP were carried out as published, using, respectively, Z-Gly-Pro-AMC and Suc-Gly-Pro-AMC as the substrate [24]. IC_50_ experiments were repeated in triplicate, and the results are presented as mean ± standard deviation. Methods and data fitting were performed as published earlier [24]. Screening against DPP4 and DPP9 was performed at final probe concentrations of 10 µM and 1 µM using Ala-Pro-*para*nitroanilide (*p*NA) as the substrate at the respective final concentrations of 25 μM (DPP4) and 150 μM (DPP9) at pH 7.4 (0.05 M HEPES-NaOH buffer with 0.1% Tween-20, 0.1 mg/mL BSA, and 150 mM NaCl). Probes were pre-incubated with the respective enzyme for 15 min at 37 °C; afterwards, the substrate was added, and the velocities of pNA release were measured kinetically at 405 nm for at least 10 min at 37 °C. Measurements were executed using the Infinite 200 (Tecan Group Ltd., Mennendorf, Switzerland), and the Magellan software was used to process the data. If the remaining activity was more than 50% at 1 μM, the IC_50_ values for the DPPs were reported as >1 µM.

### 3.5. Radiolabeling and Stability Measurements

*Gallium-68*: ^68^Ge/^68^Ga generators (ITG Garching, Germany) were used with ethanol-based post-processing evaluated by Eppard et al. [48]. Elution of gallium-68 was performed with 0.05 M HCl trapped on a micro-chromatography CEX column AG 50W-X4. The column was washed with 80% EtOH/0.15 M HCl, and ^68^Ga^3+^ was eluted with 90% EtOH/0.9 M HCl.

*Scandium-44*: Scandium-44 was obtained by a ^44^Sc/^44^Ti generator [29,30,36]. A solution of 0.005 M H_2_C_2_O_4_/0.07 M HCl was eluted through the ^44^Ti/^44^Sc generator to adsorb [^44^Sc]Sc^3+^ onto the cation exchanger AG 50 W-X8. Elution of scandium-44 was executed with 0.25 M ammonium acetate buffer pH 4.

*Lutetium-177*: n.c.a. [^177^Lu]LuCl_3_ in 0.04 M HCl was provided by ITG Garching, Germany.

Radioactivity was measured using a PC-based dose calibrator (ISOMED 2010, Nuklear Medizintechnik Dresden GmbH, Dresden, Germany). Reaction controls were done using radio-TLC, with 0.1 M citrate buffer pH 4 and an analytical HPLC 7000 series Hitachi LaChrom with a Phenomenex Luna C18 column (250 × 4.6 mm, 5 μm), linear gradient of 5–95% MeCN (+0.1% TFA)/H_2_O (+0.1% TFA), flow rate 1 mL/min in 10 min. TLCs were measured in a CR-35 Bio Test-Imager from Duerr-ndt (Bietigheim-Bissingen, Germany) with the analysis software AIDA Elysia-Raytest (Straubenhardt, Germany).

Labeling was carried out with 100–150 MBq gallium-68 in 300 µL of 1 M ammonium acetate (AmAc) buffer pH 5.5 and with 30–40 MBq scandium-44 in 1 mL of 0.25 M AmAc pH 4.0, and aliquots were taken at 1, 3, 5, 10, and 15 min. For lutetium-177, activity of 30–40 MBq in 300 µL of 1 M AmAc pH 5.5 was used, and aliquots were taken at 1, 3, 5, 15, 30, and 60 min. Stability was tested in 500 µL of human serum, phosphate-buffered saline, ethanol, and saline (0.9% isotonic NaCl solution) using ~5 MBq of tracer solution with >95% radiochemical purity. The measured time points were adjusted to the physical half-lifes, i.e., gallium-68 (15, 30, 60, 90, 120 min), scandium-44 (0.5, 1, 2, 4, 8 h), and lutetium-177 (1–6 h, 1, 2, 3, 7, 10 d). HS (human male AB plasma, USA origin) and PBS were purchased from Sigma Aldrich, and 0.9% saline from B. Braun Melsungen AG (Melsungen, Germany).

### 3.6. Lipophilicity Determination

Lipophilicity of [^68^Ga]Ga-AAZTA^5^.SA.FAPi, [^44^Sc]Sc-AAZTA^5^.SA.FAPi and [^68^Ga]Ga-DOTA.SA.FAPi was determined using the “shake-flask” methodology. After reaction of the precursor with the respective radionuclide, the reaction solution was adjusted to pH 7.4 with NaOH. Aliquots of ~5 MBq for the ^68^Ga complexes and of ~3 MBq for the ^44^Sc-complexed were taken and adjusted to a total volume of 700 μL with PBS (*n*= 4). 700 μL 1-octanol was added, and the solution was shaken for 2 min (1500 rpm). Afterwards, each tube was centrifuged for 2 min. 400 μL of the octanol- and PBS phases were pipetted in new tubes, and aliquots of each phase (3 µL of the PBS phase and 6 μL of the octanol phase) were measured via radio-TLC. The PBS phases were adjusted to 700 µL, and 700 μL octanol was added to each tube. The procedure was repeated twice. LogD values were calculated as the logarithm of the octanol/PBS ratio.

## 4. Conclusions

In this work, a new squaramide FAPi conjugate to the AAZTA chelator is introduced. After successful preparative synthesis, the complex was tested for its in vitro binding characteristics and compared to the analogue DOTA.SA.FAPi derivative, published recently [24]. The inhibitory potency studies of AAZTA^5^.SA.FAPi showed excellent sub-nanomolar affinities for FAP, in the same order of magnitude of those of the already published SA.FAPi monomeric structures DATA^5m.^SA.FAPi and DOTA.SA.FAPi. Furthermore, high selectivity against PREP and the DPPs was achieved. AAZTA^5^.SA.FAPi labeling with gallium-68, scandium-44, and lutetium-177, as well DOTA.SA.FAPi complexation with scandium-44 and lutetium-177, were successfully performed. Remarkably, for AAZTA^5^.SA.FAPi, compared to the DOTA derivative, [^44^Sc]Sc-AAZTA.SA.FAPi required significantly less precursor for quantitative labeling, resulting in higher specific activities, and performed complete complexation at ambient temperatures. The stability of the radiometal-complexed AAZTA^5^.SA.FAPi in various media was excellent, as demonstrated by the presence of highly intact conjugates. Complexation with the β^+^-emitting scandium-44 may offer a good alternative to gallium-68 usage in diagnosis due to the longer half-life of 4 h and the favorable traits of this nuclide. Interesting is also the remarkable labeling and stability with lutetium-177, allowing therapeutical application. A first theranostic approach of DOTA.SA.FAPi was reported by Ballal et al. [49]. Therefore, the new FAP inhibitor-based probes DOTA.SA.FAPi and AAZTA^5^.SA.FAPi, complexed with gallium-68, scandium-44, and lutetium-177, are promising radiopharmaceuticals for use in a theranostic settings.

## Figures and Tables

**Table 1:** IC_50_ values of AAZTA^5^.SA.FAPi and DOTA.SA.FAPi derivatives for FAP and the related proteases DPPs and PREP. Data are presented as the mean with standard deviation (*n* = 3 for FAP and PREP and *n* = 2 for the DPPs).

Compound	DPPs IC_50_ (µM)	PREP IC_50_ (µM)	FAP IC_50_ (nM)	Selectivitiy Index (FAP/PREP)
AAZTA^5^.SA.FAPi	>1	2.4 ± 0.4	0.56 ± 0.02	4286
[^nat^Sc]Sc-AAZTA^5^.SA.FAPi	>1	3.6 ± 0.8	0.57 ± 0.04	6316
[^nat^Lu]Lu-AAZTA^5^.SA.FAPi	>1	3.2 ± 0.6	0.55 ± 0.04	5818
DOTA.SA.FAPi	n.d.	5.4 ± 0.3 ^a^	0.9 ± 0.1 ^a^	6000
[^nat^Ga]Ga-DOTA.SA.FAPi	>1	8.7 ± 0.9 ^a^	1.4 ± 0.2 ^a^	6214
[^nat^Lu]Lu DOTA.SA.FAPi	>1	2.5 ± 0.4 ^a^	0.8 ± 0.2 ^a^	3125
DATA^5m^.SA.FAPi	n.d.	1.7 ± 0.1 ^a^	0.8 ± 0.2 ^a^	2113
[^nat^Ga]Ga-DATA^5m^.SA.FAPi	>1	4.7 ± 0.3 ^a^	0.7 ± 0.1 ^a^	6714
UAMC1110-FAP inhibitor	>10	1.8 ± 0.01 ^b^	0.43 ± 0.07 ^a^	4186

^a^ data from Moon et al. [24]; ^b^ data from Jansen et al. [12]; n.d. not determined.

**Table 2:** LogD values (pH = 7.4) of [^68^Ga]Ga-AAZTA^5^.SA.FAPi, [^44^Sc]Sc-AAZTA^5^.SA.FAPi and [^68^Ga]Ga-DOTA.SA.FAPi.

Compound	LogD_7.4_
[^68^Ga]Ga-AAZTA^5^.SA.FAPi	−2.53 ± 0.13
[^44^Sc]Sc-AAZTA^5^.SA.FAPi	−2.50 ± 0.11
[^68^Ga]Ga-DOTA.SA.FAPi	−2.68 ± 0.06

## Data Availability

The study did not report any data.

## Supplementary Materials

Figure S1: radio-HPLC spectra of [^68^Ga]Ga-AAZTA^5^.SA.FAPi after reaction of 15 min with linear gradient condition of 10–95% MeCN (+0.1% TFA)/95–10% water (+0.1% TFA) in 10 min, 1 mL/min, t*_R_* = 8.4 min; Figure S2: Stability test: radio-HPLC spectra of [^68^Ga]Ga-AAZTA^5^.SA.FAPi in ethanol after 1 h with linear gradient condition of 5–95% MeCN (+0.1% TFA)/95–5% water (+0.1% TFA) in 10 min, 1 mL/min, t*_R_* = 9.1 min; Figure S3: Stability test: radio-HPLC spectra of [^68^Ga]Ga-AAZTA^5^.SA.FAPi in saline after 1 h with linear gradient condition of 5–95% MeCN (+0.1% TFA)/95–5% water (+0.1% TFA) in 10 min, 1 mL/min, t*_R_* = 9.1 min; Figure S4: Stability test: radio-HPLC spectra of [^68^Ga]Ga-AAZTA^5^.SA.FAPi in human serum after 1 h with linear gradient condition of 5–95% MeCN (+0.1% TFA)/95–5% water (+0.1% TFA) in 10 min, 1 mL/min, t*_R_* = 9.1 min; Figure S5: radio-HPLC spectra of [^44^Sc]Sc-AAZTA^5^.SA.FAPi after a 15 min reaction with linear gradient condition of 5–95% MeCN (+0.1% TFA)/95-5% water (+0.1% TFA) in 10 min, 1 mL/min, t_R_ = *8*.*9* min; Figure S6: Stability test: radio-HPLC spectra of [^44^Sc]Sc-AAZTA^5^.SA.FAPi in phosphate-buffered saline after 1 h with linear gradient condition of 5–95% MeCN (+0.1% TFA)/95–5% water (+0.1% TFA) in 10 min, 1 mL/min, t*_R_* = 9.3 min; Figure S7: Stability test: radio-HPLC spectra of [^44^Sc]Sc-AAZTA^5^.SA.FAPi in saline after 1 h with linear gradient condition of 5–95% MeCN (+0.1% TFA)/95–5% water (+0.1% TFA) in 10 min, 1 mL/min, t*_R_* = 9.1 min; Figure S8: Stability test: radio-HPLC spectra of [^44^Sc]Sc-AAZTA^5^.SA.FAPi in human serum after 1 h with linear gradient condition of 5–95% MeCN (+0.1% TFA)/95–5% water (+0.1% TFA) in 10 min, 1 mL/min, t*_R_* = 9.1 min; Figure S9: Stability test: radio-HPLC spectra of [^44^Sc]Sc-AAZTA^5^.SA.FAPi in human serum after 4 h with linear gradient condition of 5–95% MeCN (+0.1% TFA)/95–5% water (+0.1% TFA) in 10 min, 1 mL/min, t*_R_* = 9.5 min; Figure S10: Stability test: radio-HPLC spectra of [^44^Sc]Sc-AAZTA^5^.SA.FAPi in saline after 4 h with linear gradient condition of 5–95% MeCN (+0.1% TFA)/95–5% water (+0.1% TFA) in 10 min, 1 mL/min, t*_R_* = 9.3 min; Figure S11: Stability test: radio-HPLC spectra of [^44^Sc]Sc-AAZTA.SA.FAPi in phosphate-buffered saline after 4 h with linear gradient condition of 5–95% MeCN (+0.1% TFA)/95–5% water (+0.1% TFA) in 10 min, 1 mL/min, t*_R_* = 9.1 min; Figure S12: radio-HPLC spectra of [^177^Lu]Lu-AAZTA^5^.SA.FAPi after a 60 min. reaction with linear gradient condition of 5–95% MeCN (+0.1% TFA)/95–5% water (+0.1% TFA) in 10 min, 1 mL/min, t*_R_* = 9.1 min.
