# Supplementary material for: In Vitro Evaluation of the Squaramide-Conjugated Fibroblast Activation Protein Inhibitor-Based Agents AAZTA5.SA.FAPi and DOTA.SA.FAPi
Source: Molecules. 2021 Jun 8;26(12):3482. doi: 10.3390/molecules26123482 (PMC8226449; doi:10.3390/molecules26123482)
Supplement: Supplementary file 1 [file molecules-26-03482-s001.zip › molecules-1186608-SI.pdf]

SUPPORTING INFORMATION

In Vitro Evaluation of the Squaramide-Conjugated Fibroblast Activation Protein Inhibitor-Based Agents AAZTA^5.SA.FAPi and DOTA.SA.FAPi

Euy Sung Moon[1], Yentl Van Rymenant[2], Sandeep Battan[1], Joni De Loose[2], An Bracke[2], Pieter Van der Veken[3], Ingrid De Meester[2], Frank Rösch[1]*

[1] Department of Chemistry – TRIGA, Johannes Gutenberg University Mainz, 55128 Mainz, Germany; emoon01@uni-mainz.de
[2] Department of Pharmaceutical Sciences, Laboratory of Medical Biochemistry, University of Antwerp, 2610 Wilrijk, Belgium; ingrid.demeester@uantwerpen.be
[3] Department of Pharmaceutical Sciences, Laboratory of Medicinal Chemistry, University of Antwerp, 2610 Wilrijk, Belgium; pieter.vanderveken@uantwerpen.be
* Correspondence: frank.roesch@uni-mainz.de; Tel.: +4961313925302

Radiolabeling

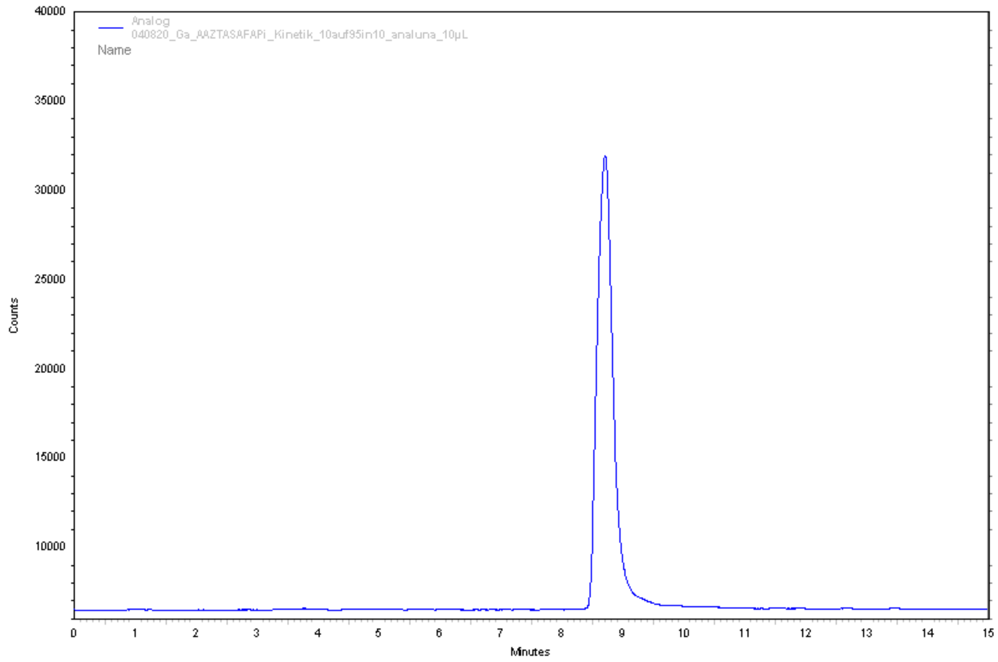

Figure S1: radio-HPLC spectra of [^{68}Ga]Ga-AAZTA^5.SA.FAPi after reaction of 15 min. with linear gradient condition of 10-95 % MeCN (+0.1 % TFA)/95-10 % Water (+0.1 % TFA) in 10 min, 1 mL/min, t_R= 8.4 min.

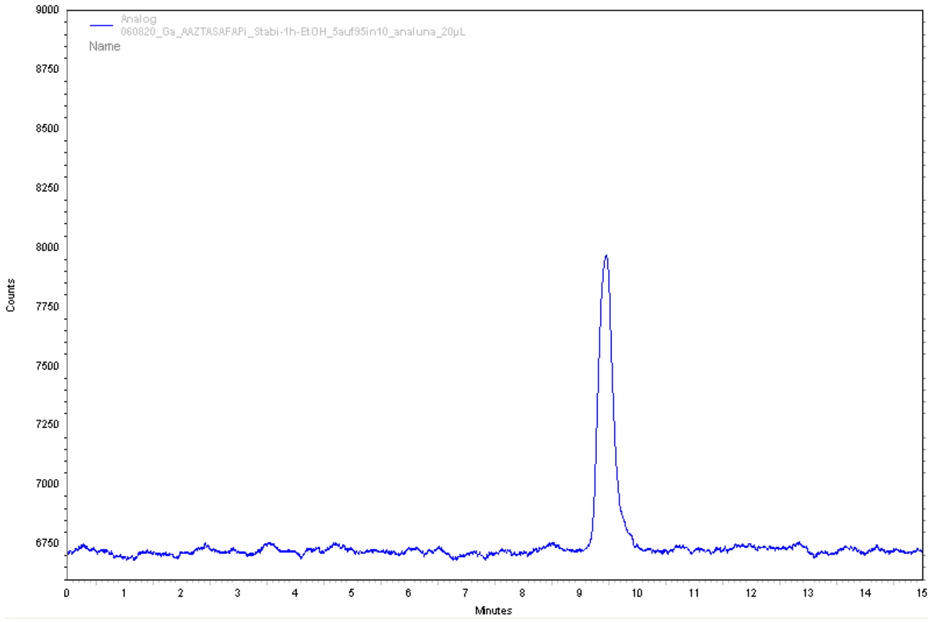

Figure S2: Stability test: radio-HPLC spectra of [^{68}Ga]Ga-AAZTA^5.SA.FAPi in Ethanol after 1 h with linear gradient condition of 5-95 % MeCN (+0.1 % TFA)/95-5 % Water (+0.1 % TFA) in 10 min, 1 mL/min, t_R = 9.1 min.

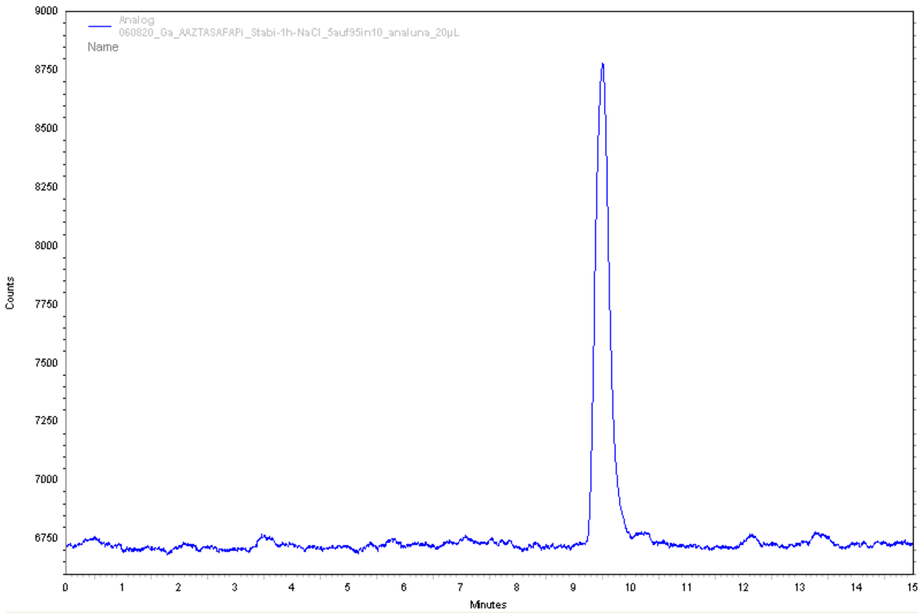

Figure S3: Stability test: radio-HPLC spectra of [^{68}Ga]Ga-AAZTA^{5}.SA.FAPi in saline after 1 h with linear gradient condition of 5-95 % MeCN (+0.1 % TFA)/95-5 % Water (+0.1 % TFA) in 10 min, 1 mL/min, t_R = 9.1 min.

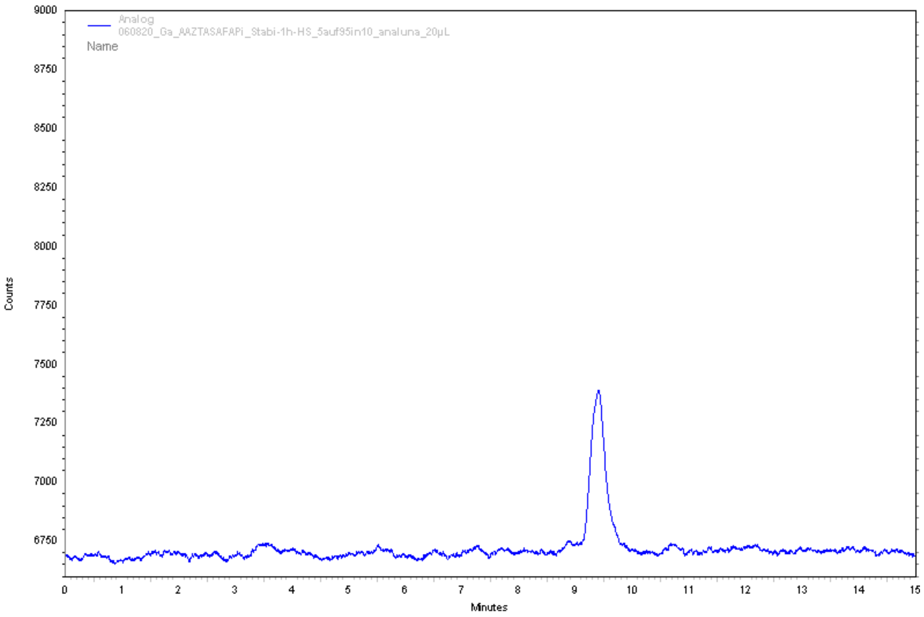

Figure S4: Stability test: radio-HPLC spectra of [^{68}Ga]Ga-AAZTA^{5}.SA.FAPi in human serum after 1 h with linear gradient condition of 5-95 % MeCN (+0.1 % TFA)/95-5 % Water (+0.1 % TFA) in 10 min, 1 mL/min, t_R = 9.1 min.

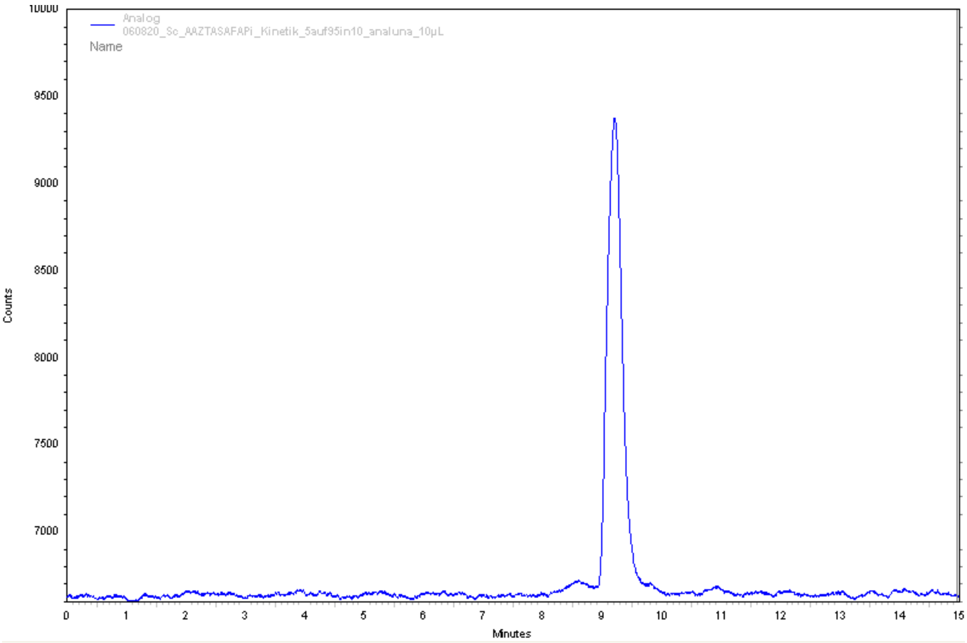

Figure S5: radio-HPLC spectra of [^{44}Sc]Sc-AAZTA^{5}.SA.FAPi after 15 min. reaction with linear gradient condition of 5-95 % MeCN (+0.1 % TFA)/95-5 % Water (+0.1 % TFA) in 10 min, 1 mL/min, t_R = 8.9 min.

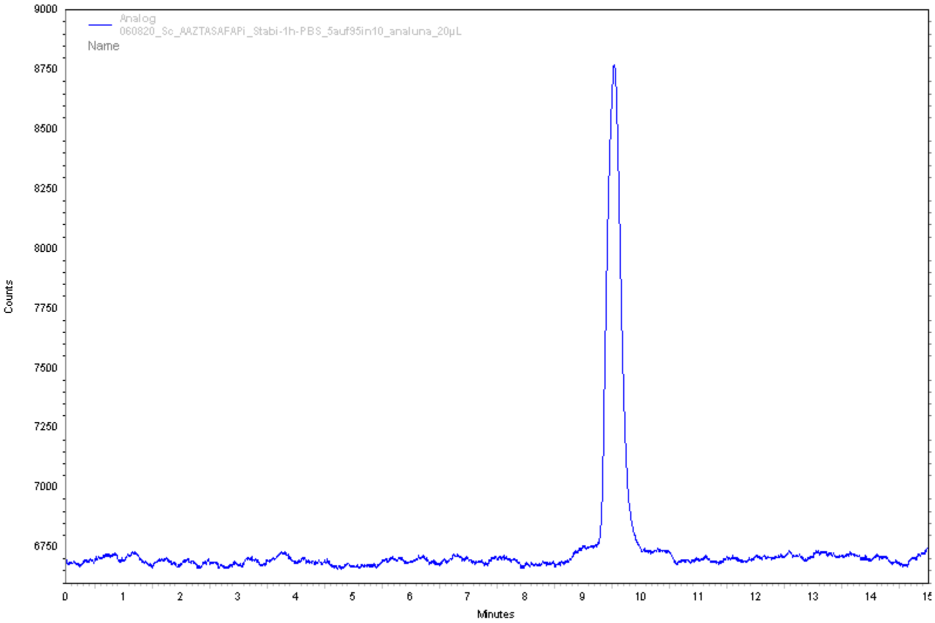

Figure S6: Stability test: radio-HPLC spectra of [^{44}Sc]Sc-AAZTA^{5}.SA.FAPi in phosphate buffered saline after 1 h with linear gradient condition of 5-95 % MeCN (+0.1 % TFA)/95-5 % Water (+0.1 % TFA) in 10 min, 1 mL/min, t_R = 9.3 min.

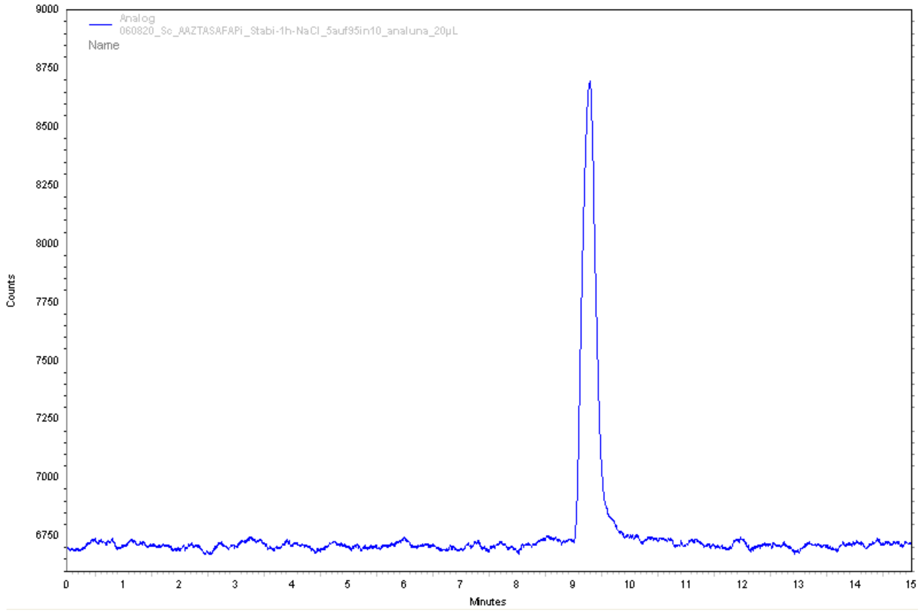

Figure S7: Stability test: radio-HPLC spectra of [44Sc]Sc-AAZTA5.SA.FAPi in saline after 1 h with linear gradient condition of 5-95 % MeCN (+0.1 % TFA)/95-5 % Water (+0.1 % TFA) in 10 min, 1 mL/min, t_R = 9.1 min.

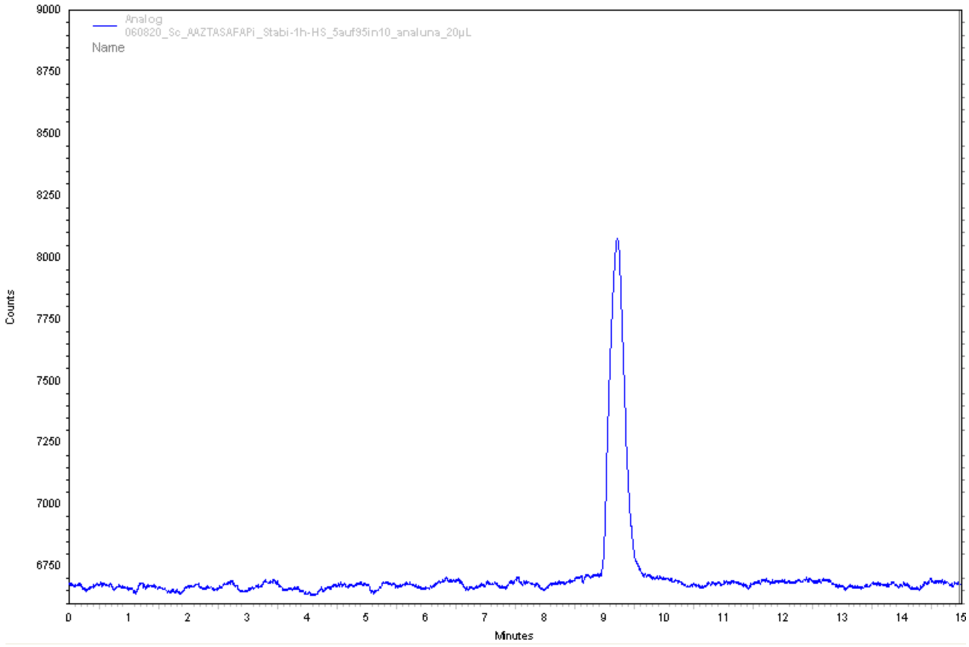

Figure S8: Stability test: radio-HPLC spectra of [44Sc]Sc-AAZTA5.SA.FAPi in human serum after 1 h with linear gradient condition of 5-95 % MeCN (+0.1 % TFA)/95-5 % Water (+0.1 % TFA) in 10 min, 1 mL/min, t_R = 9.1 min.

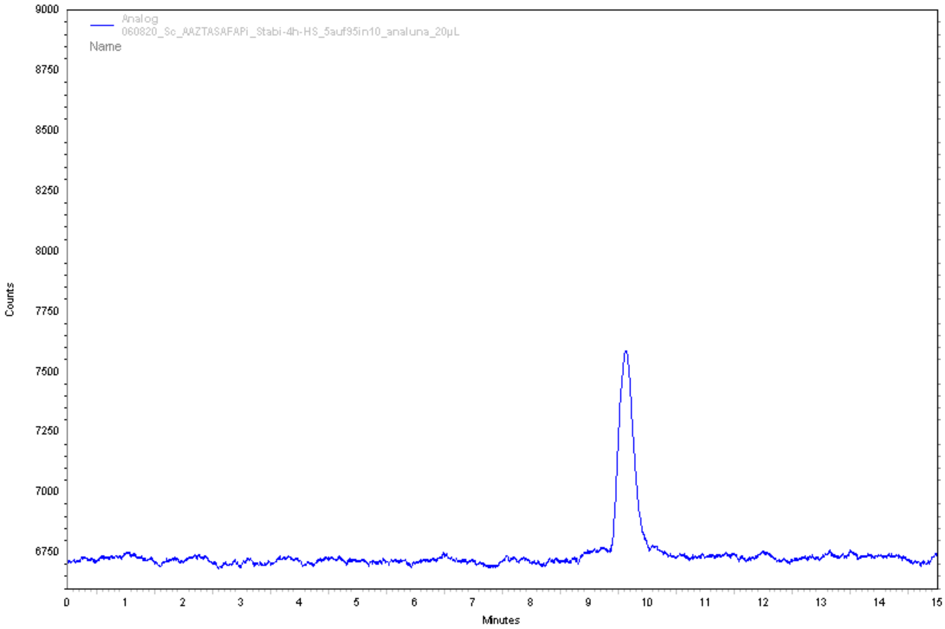

Figure S9: Stability test: radio-HPLC spectra of [^{44}Sc]Sc-AAZTA^5.SA.FAPi in human serum after 4 h with linear gradient condition of 5-95 % MeCN (+0.1 % TFA)/95-5 % Water (+0.1 % TFA) in 10 min, 1 mL/min, t_R = 9.5 min.

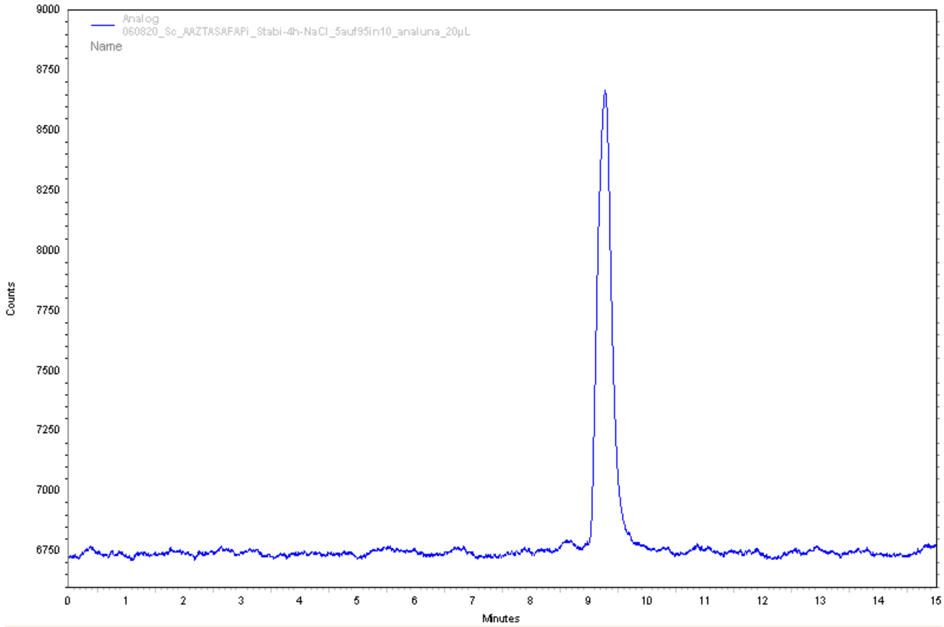

Figure S10: Stability test: radio-HPLC spectra of [^{44}Sc]Sc-AAZTA^5.SA.FAPi in saline after 4 h with linear gradient condition of 5-95 % MeCN (+0.1 % TFA)/95-5 % Water (+0.1 % TFA) in 10 min, 1 mL/min, t_R = 9.3 min.

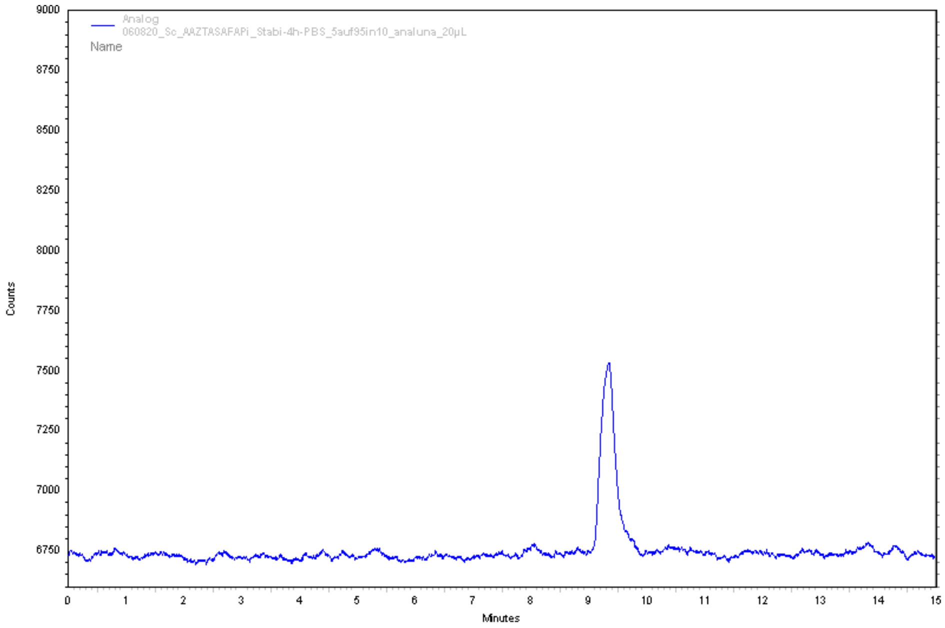

Figure S11: Stability test: radio-HPLC spectra of [^{44}Sc]Sc-AAZTA^{5}.SA.FAPi in phosphate buffered saline after 4 h with linear gradient condition of 5-95 % MeCN (+0.1 % TFA)/95-5 % Water (+0.1 % TFA) in 10 min, 1 mL/min, t_R = 9.1 min.

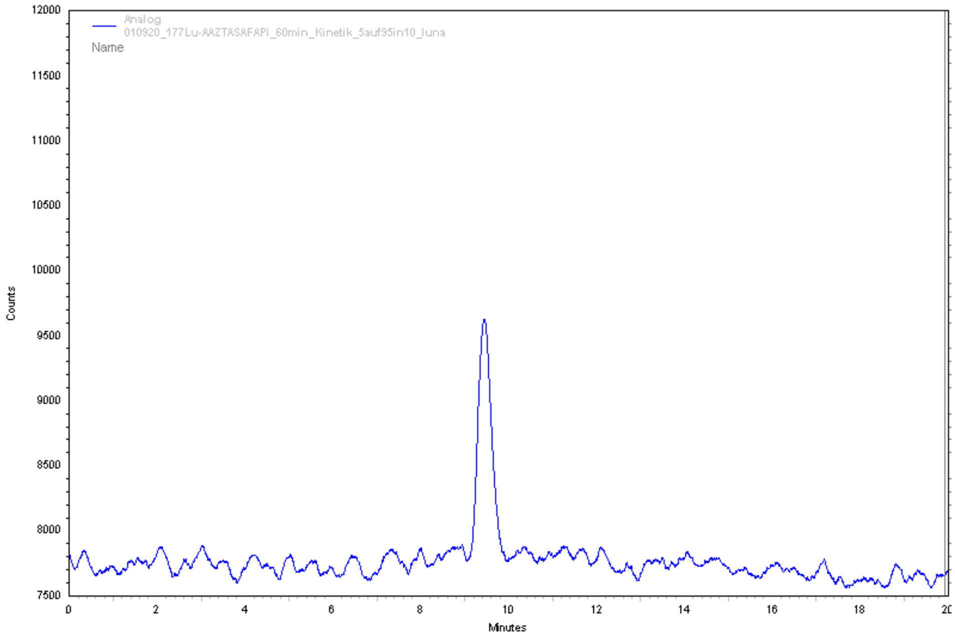

Figure S12: radio-HPLC spectra of [^{177}Lu]Lu-AAZTA^{5}.SA.FAPi after 60 min. reaction with linear gradient condition of 5-95 % MeCN (+0.1 % TFA)/95-5 % Water (+0.1 % TFA) in 10 min, 1 mL/min, t_R = 9.1 min.
